# The Influence of Media Diversification Model and Entrepreneurship on Enterprise Financial Performance Under the Environment of Sustainable Development

**DOI:** 10.3389/fpsyg.2022.885452

**Published:** 2022-06-20

**Authors:** Xinying Li, Shuaifu Lou, Huiqin Zhu

**Affiliations:** Department of Management, Jeonju University, Jeonju, South Korea

**Keywords:** sustainable development, media diversification, entrepreneurship, financial performance, diversified operation mode

## Abstract

Market competition is intensifying. The necessity and path of adopting the diversified management model in the media industry are explored to delve into the influence of the media diversification model and entrepreneurship on enterprise financial performance. Besides, the relevant theories such as the media diversification model and entrepreneurial spirit are expounded. Furthermore, Time Publishing & Media is taken as the representative of the media diversification model. Finally, the influence of entrepreneurship on financial performance is discussed regarding entrepreneurship in the Yangtze River Delta as the research object. The profitability, solvency, and operation ability of Time Publishing & Media are analyzed. It is found that there are problems in the profitability and operation ability of Time Publishing & Media. The solvency is good, and the risk of debt repayment is low. As a result, a diversified management model may not have a positive impact on enterprise performance. In addition, the entrepreneurial spirit of the Yangtze River Delta is studied, and the results reveal that the *F* values from 2017 to 2019 are about 66.24, 10.78, and 60.39, respectively, with a significance of 0.00. It implies that the stronger the entrepreneur’s ability to take risks, the better the financial performance of the enterprise, but the risk should be appropriate. Therefore, the research on the influence of the media diversification model and entrepreneurship on the financial performance of enterprises in the environment of sustainable development has guided significance for enterprises to improve their business performance and market competitiveness.

## Introduction

With the continuous improvement of the economic level, many enterprises have appeared in the market. The continuous enrichment of the market has brought new challenges to enterprises. Financial performance is an indicator to measure the operational capability of an enterprise. It fully expresses the effect of cost control, asset utilization and management, capital allocation, and the composition of the return on shareholders’ equity (Bhattarai et al., 2019). The theory of sustainable development requires enterprises to promote the harmonious development of the economy, nature, and society while improving efficiency. Therefore, how to improve financial performance has increasingly become one of the key concerns of enterprises under the coexistence between man and nature (Li et al., 2021).

After interviewing 100 successful local entrepreneurs about financial performance, Hong et al. (2018) used a series of equations to obtain enterprise valuation data. Secondly, they found that the media diversification model brought about 10%–15% of losses to the enterprise by comparing the estimated results with the real results (Hong et al., 2018). Achi et al. (2021) analyzed the financial data of 500 Japanese companies using empirical research from the perspective of sustainable development. They finally found that diversified management methods could not improve the profitability of the enterprises, but the performance of such methods was significantly lower than that of specialized management enterprises. Meanwhile, the diversification of enterprises also reduced financial performance. In addition, the more industries a company invests in, the greater the decline in the company’s performance (Achi et al., 2021). Nhemachena and Murimbika (2018) conducted an empirical study on the financial data of 250 Fortune 500 companies and found that with the deepening of their diversification, enterprise performance showed a trend of first rising and then falling. The result showed that diversification did not simply generate performance premium or performance discount, but there was a critical point (Nhemachena and Murimbika, 2018). Chen (2019) used data meta-analysis to study the relationship between diversification and performance and took market performance as the evaluation content of enterprise performance. The results showed that the relationship between enterprise diversification and performance was not just a simple linear, but an inverted U-shaped curve. This indicated that the performance of the enterprise first increased and then decreased with the deepening of diversification (Chen, 2019). Luchko and Zinkevych (2019) believed that entrepreneurship was composed of a spirit of the contract, risk, and excellence, which leveraged the progress of society (Luchko and Zinkevych, 2019). Pan et al. (2020) took the Yangtze River Delta as the research object and used a questionnaire survey as a research method. A structural equation model was established using organizational learning, organizational innovation, and enterprise performance based on the theory of sustainable development. Finally, it was concluded that entrepreneurship was achieved through two intermediate variables of organizational learning and organizational innovation, which indirectly affected enterprise performance (Pan et al., 2020). The theories and methods of the above-mentioned experts and scholars have provided help in the actual development and operation. These concepts have brought methodological guidance for the improvement of corporate financial performance to varying degrees. However, they mostly start from a single point of view, such as valuation data, performance premium, profitability, and entrepreneurial spirit. Given this, this paper will evaluate the overall operation ability of Time Publishing & Media, which is comprehensive.

**Figure 1:**
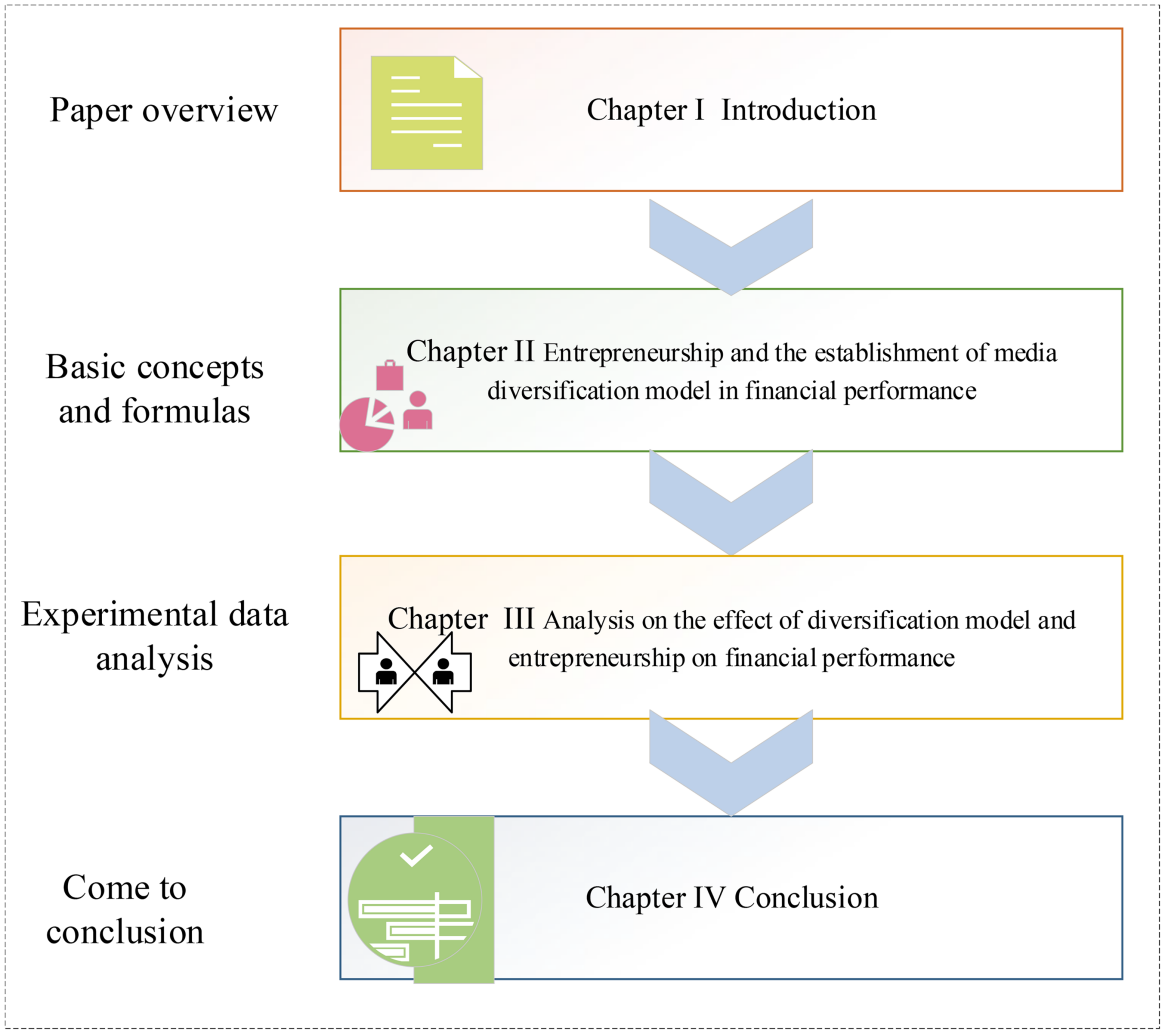
Organizational structure.

To sum up, the relationship between media diversification, entrepreneurship, and financial performance is analyzed under the environment of sustainable development. Then, Time Publishing & Media and some enterprises in the Yangtze River Delta are taken as the research objects, and how the media diversification model and entrepreneurship affect the financial performance of enterprises is expounded. The innovation lies in that it is logical to use the methods of questionnaire survey and comparative analysis to study the operating capacity, solvency, and profitability of Time Publishing & Media. In addition, discussing the risk-taking ability of entrepreneurs in the Yangtze River Delta in recent years helps subsequent entrepreneurs. The purpose of this paper is to provide a methodological reference for improving the performance of enterprises and promoting the long-term development of enterprises. The organizational structure of this paper is shown in Figure 1.

## Entrepreneurship and the Establishment of the Media Diversification Model in Financial Performance

### Media Diversified Management Model and Entrepreneurship

**Figure 2:**
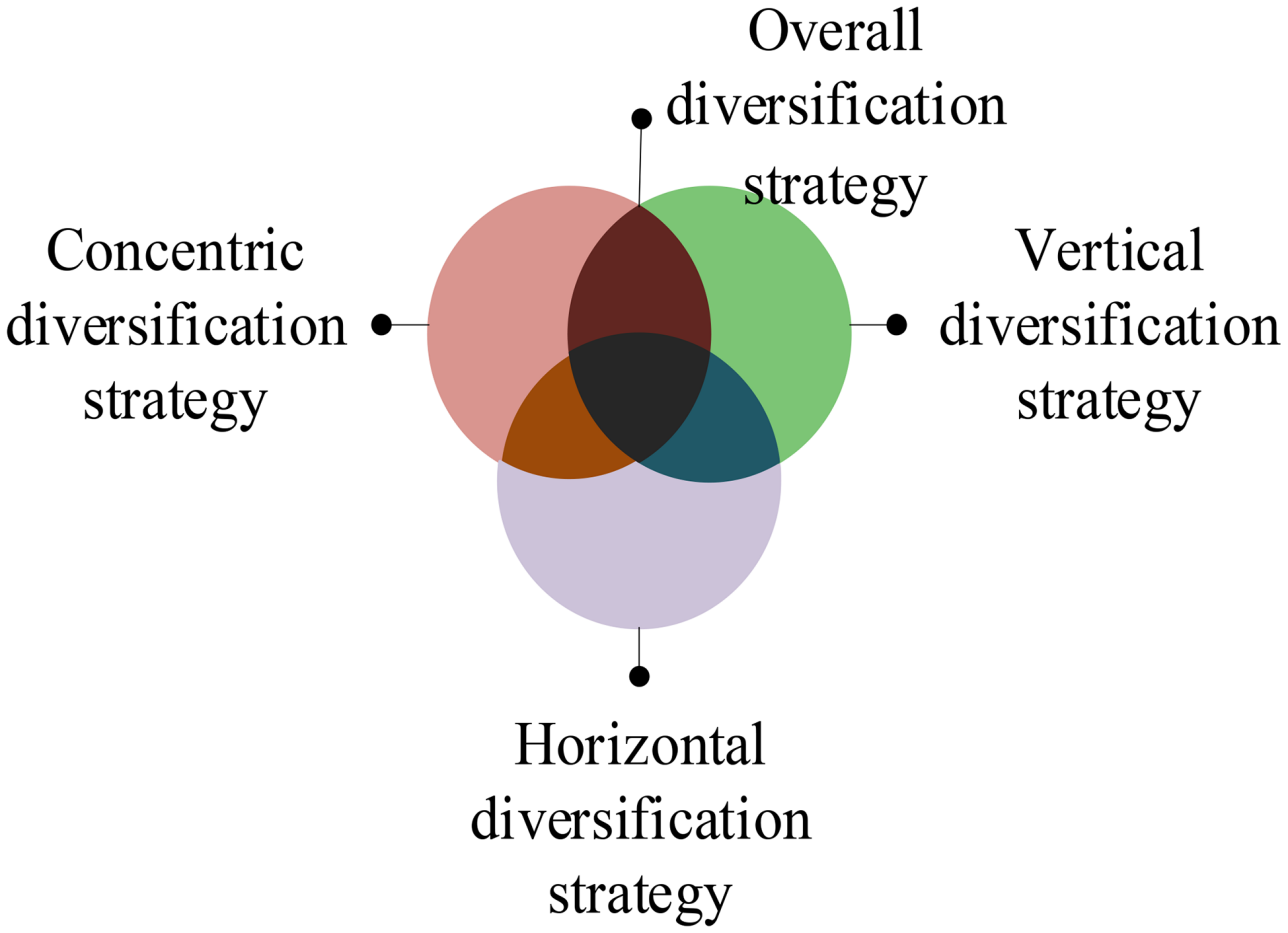
Types of enterprise diversification.

The diversified operation mode means that the enterprise’s operation is not limited to one product or industry, but implements cross-product and cross-industry operation expansion. The business model belongs to the development strategy and is a long-term plan for the enterprise to develop multiple varieties or diversified operations (Zheng et al., 2018; Pinheiro et al., 2021). Diversification is mainly aimed at the types and quantity of products operated by enterprises. However, it is not accurate to define the diversification of enterprises by the number of product types. The reason is that highly related multi-product operations and highly unrelated cross-industry multi-product operations show different degrees of diversification, even if the number of final product types of enterprises is the same. The latter has a higher degree of diversification and a greater impact on enterprise operation (Krosnick et al., 2018; Azeem et al., 2020). There are various modes of enterprise diversification, which can be summarized into the following four types, as shown in Figure 2.

From Figure 1, the types of enterprise diversification include concentric diversification strategy, horizontal diversification strategy, vertical diversification strategy, and overall diversification strategy. The concentric diversification strategy means that the enterprise uses the original production technology to manufacture new products with different uses from the original products. The horizontal diversification strategy means that the enterprise produces new products and sells them to the customers in the original market to meet their new needs. The vertical diversification strategy is also known as a longitudinal diversification strategy. It is further divided into forwarding integrated business strategy and backward integrated business strategy. The overall diversification strategy refers to the expansion of the enterprise to the business scope unrelated to the original product, technology, and market. The measurement of enterprise diversification can make managers understand the dispersion of enterprise diversification funds clearly to make the most effective strategic deployment. At present, the commonly used diversification measurement methods are Herfindahl–Hirschman Index (HDI) and entropy measurement method (EDI; Min and Kim, 2021).

Herfindahl–Hirschman Index is a measure of market concentration. It refers to the square sum of the income of market competitors in the industry as a percentage of the total industry income. When measuring enterprise diversification with this index, *X_i_* represents the income of the i-th product or business of the enterprise, *X* represents the total income of the enterprise, and *n* reflects the product quantity or business quantity of the enterprise. When the value of HDI is closer to 1, the enterprise shows professional operation. When the value is lower than 0.5, the enterprise has a high degree of diversification. At this time, the degree of resource dispersion is high, and the risk increases gradually (Ganiyu et al., 2020). Equation (1) is the HDI calculation method.


(1) $$HDI=\overset{n}{\underset{i=1}{\sum }}{\left(X_{i}/X\right)}^{2}$$

Entropy measurement method is proposed by Palepu, which borrows the concept of entropy in information theory and has the meaning of average information (Subramanian et al., 2019). Equation (2) is the specific calculation method.


(2) $$EDI=\overset{n}{\underset{i=1}{\sum }}{\left(1/X_{i}\right)}^{2}$$

The meaning of letters in Equation (2) is the same as the above equation. When EDI is larger, enterprise diversification is higher. The smaller the EDI is, the lower the degree of enterprise diversification is. Compared with HDI, EDI data calculation is more difficult. However, they both have a better theoretical background and practical value in general.

**Figure 3:**
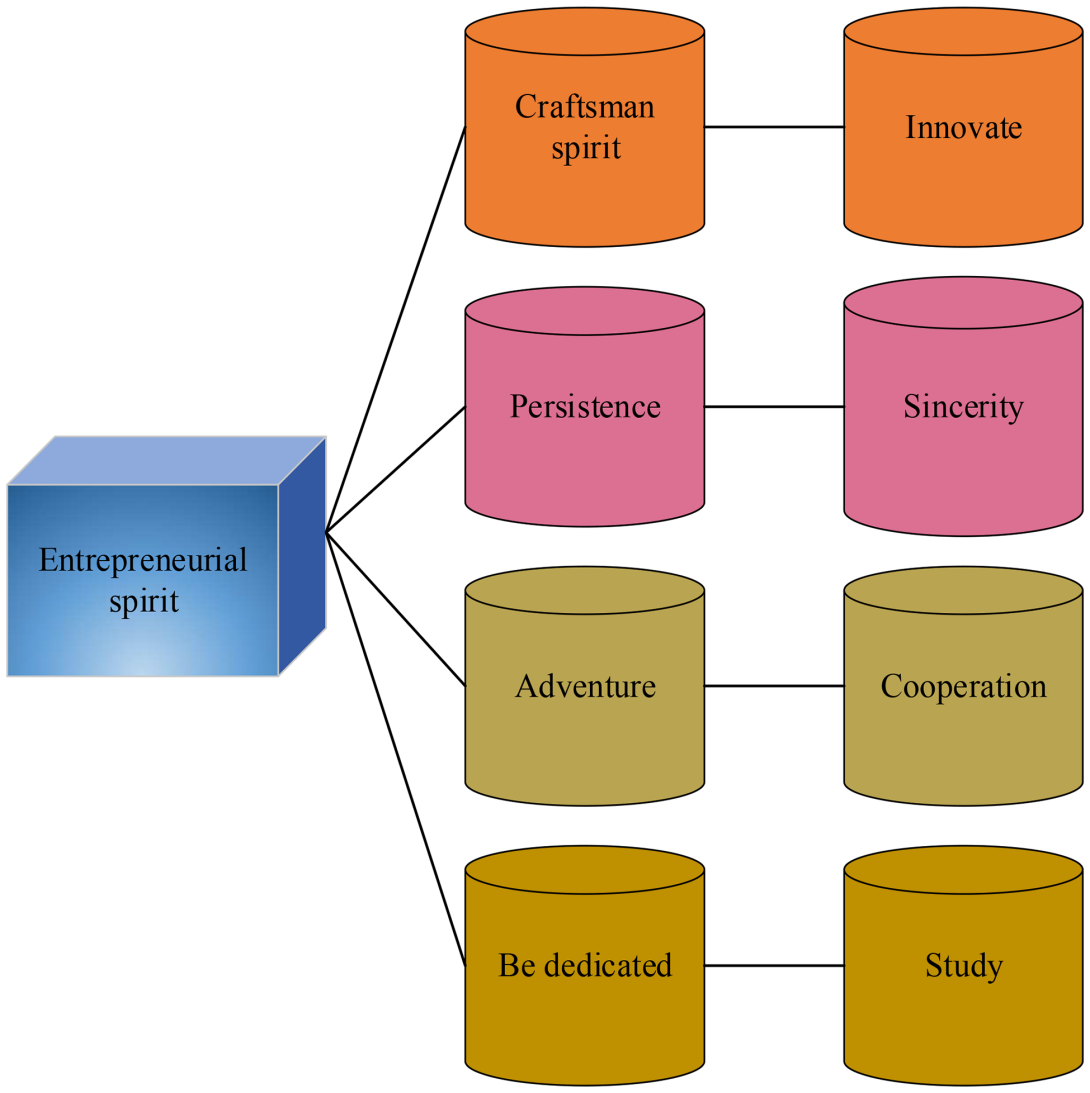
Characteristics of true entrepreneurship.

Entrepreneur refers to the general manager or chairman who directly manages the enterprise, allocates enterprise resources, affects the major business decisions of the enterprise, plays a key role in the development of the enterprise, and can affect the future and destiny of the enterprise to a great extent (Amun et al., 2018). Entrepreneurship refers to the special ability of some people to organize land, labor, capital, and other resources to produce goods, find new business opportunities, and develop new business models (Wenke et al., 2021). True entrepreneurship has the following characteristics. Figure 3 is the specific content.

Real entrepreneurship can be concluded as entrepreneurs should first have the spirit of craftsmanship; innovation is the soul of entrepreneurship; adventure is the nature of entrepreneurship; cooperation is the quality of entrepreneurship; dedication is the driving force of entrepreneurship; learning is the key to entrepreneurship; perseverance is the essence of entrepreneurship; and integrity is the cornerstone of entrepreneurship.

### The Necessity and Path of Diversified Management of Media Enterprises

(1) Different from other enterprises, media enterprises have their unique nature. The particularity of this industry promotes its diversified operation to a certain extent. First, the business contents of media enterprises include books, newspapers, textbooks, and teaching aids. These communication contents are related to culture, so they transmit culture. The business of media enterprises is mainly to provide cultural creativity for the market through the processing and dissemination of information, so the media industry also has the attribute of the service industry. Next, the products and services of the media industry do not have price elasticity (Lee et al., 2020). The demand for cultural products and services is relatively inelastic and insensitive to price changes. People’s demand will not rise significantly due to the decline in product and service prices. Therefore, it is difficult for media enterprises to realize the growth of enterprise income by reducing prices. The ownership and use rights of media are separated from each other. Due to the cultural nature of the media industry, the state has strict control over the media, and its ownership belongs to the state. However, the use right belongs to state-owned enterprises or private enterprises. The state issues relevant policies to guide the development of the media industry, which has certain constraints. Therefore, the development of media enterprises is greatly affected by national policies and systems (Iwara et al., 2020). (2) In recent years, with the government’s proposal to revitalize the cultural industry, deepen the cultural system reform, and stimulate the cultural creativity of the whole society, the competition in the media industry has become increasingly fierce (Xie, 2021). Therefore, implementing a diversified management strategy is the best choice for publishing and media enterprises. (3) With the rapid progress of science and technology, Internet coverage is extensive, and new communication modes have emerged in the media industry (Meekaewkunchorn et al., 2021). Although the information authenticity of the original media is relatively high, the new media have more advantages in communication speed, communication mode, and communication content. Therefore, people prefer to obtain information through new media (Kebede, 2020).

**Figure 4:**
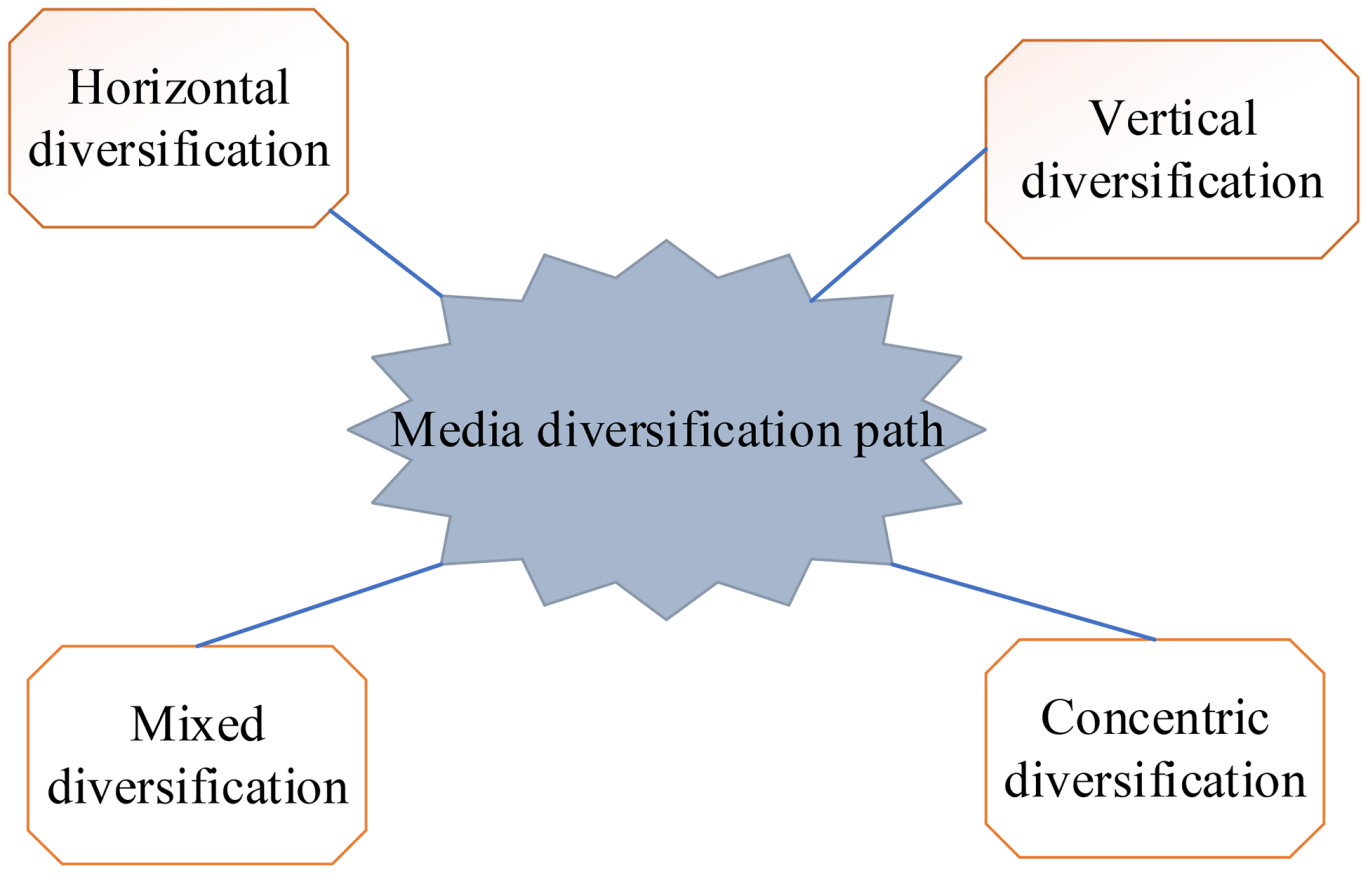
The business path of media diversified enterprises.

At present, the diversified operation has been the primary choice of media enterprises. The main business fields can be divided into business related to the publishing industry and business unrelated to the publishing industry (Hoang and Phi, 2020). Figure 4 shows four specific business paths.

From Figure 4, the operating paths of media diversification enterprises are divided into horizontal diversification, vertical diversification, mixed diversification, and concentric diversification. Horizontal diversification means that companies produce new products and sell them to customers in the original market to meet their new needs. For example, a food machine company originally produces food machines to sell to food processing plants and harvesters to farmers. Later, it produces agrochemicals, which are still sold to farmers. The vertical diversification business model includes forward integrated operation and backward integrated operation. Mixed diversification refers to the expansion of enterprises to business scope unrelated to original products, technology, and market. For example, the main business of the American Telephone & Telegraph is telecommunications, and it expands into the hotel business later. Concentric diversification means that enterprises use the original production technology conditions to manufacture new products with different uses from the original products. For example, automobile factories produce automobiles, tractors, and diesel engines.

### The Relationship Between Diversification and Financial Performance of Media Enterprises

**Figure 5:**
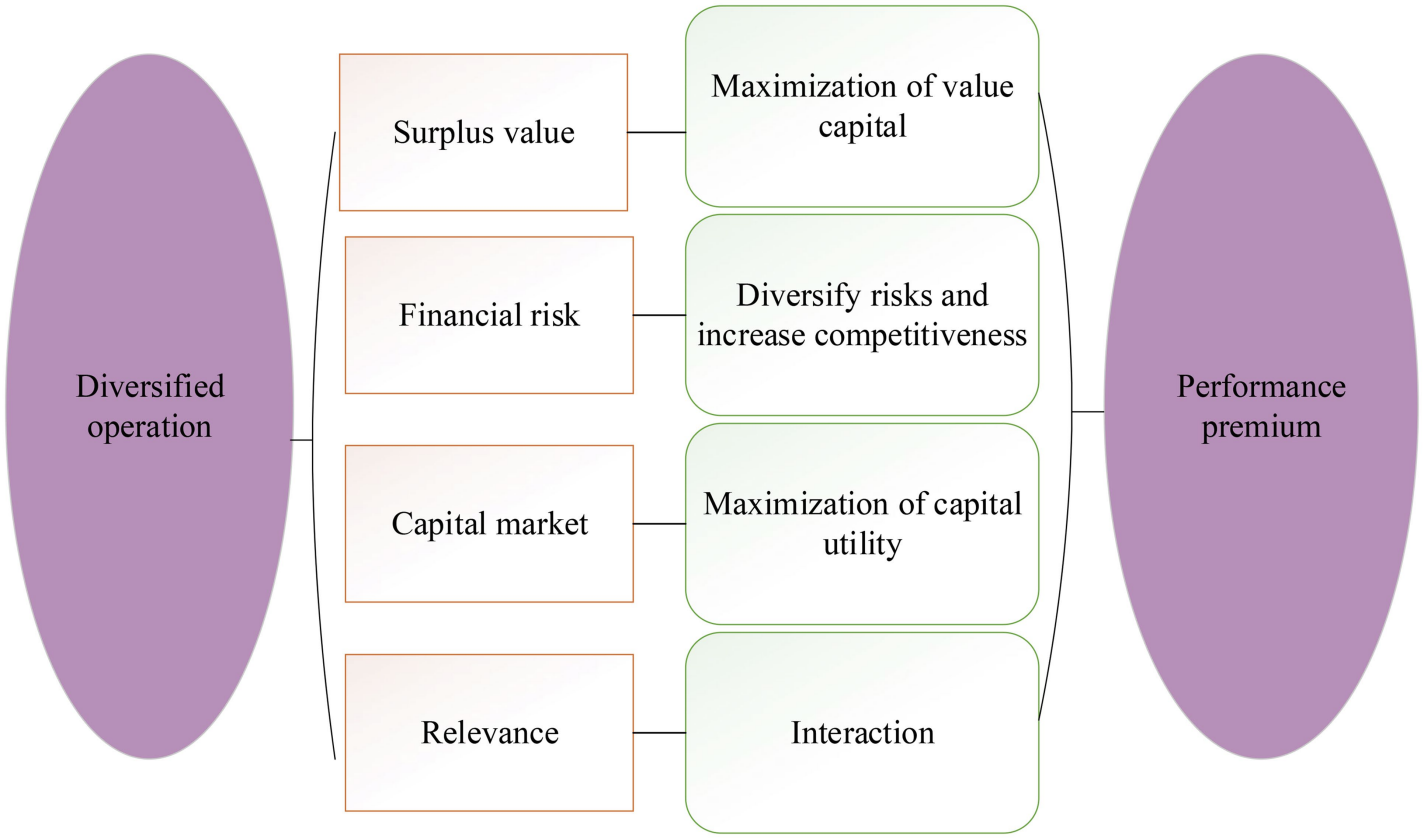
Positive impact of diversification.

The diversified business model can bring opportunities for the development of enterprises, but it also hinders their economic improvement. The positive and negative effects of diversification were analyzed. Figure 5 displays the positive impact.

Figure 5 indicates that media enterprises can optimize the allocation of resources and improve the utilization efficiency of remaining resources among various operating units by adopting a media diversification model. Meanwhile, the funds of the enterprise can be dispersed into different fields due to the particularity of the media diversification model. It also effectively diversifies the business risk of the enterprise. Moreover, an enterprise’s diversified management model often involves multiple industries. There are differences in the speed and time of return for different investments. So the internal capital market is established (Kusa et al., 2021; Mesaros et al., 2021). Fund allocation between different businesses can also reduce idle funds and improve the capital utilization rate and return on investment of enterprises. Financial synergies are achieved, and enterprise performance is improved (Wen, 2020). In addition, vertical integration reduces the intermediate links in commodity circulation. Diversified operations expand the business scope, realize transaction internalization, and reduce transaction difficulty and cost. Also, the decline in transaction costs leads to a decline in the selling price of commodities and a reduction in the spillover of marginal profits, thereby enhancing the enterprise’s ability to collect payments and forming a competitive advantage.

**Figure 6:**
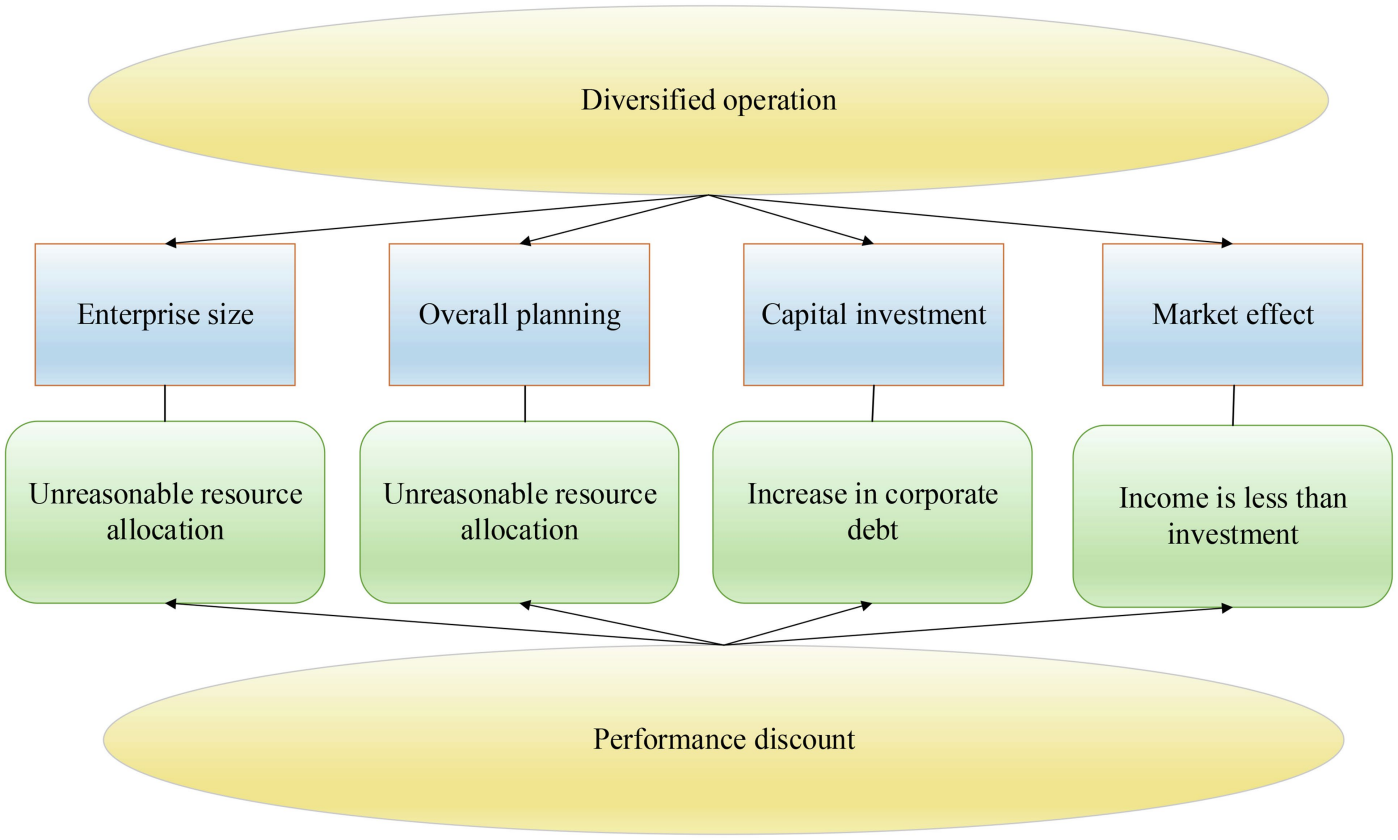
The negative effects of diversification.

The negative effects of diversification are shown in Figure 6.

According to Figure 6, a diversified enterprise needs to allocate limited resources. The unreasonable allocation of resources will lead to the dispersion of resources and reduce the utilization of resources and the ability of resources to create value. Therefore, the development of enterprises will be impeded (Hanggraeni et al., 2019). Besides, media diversification will increase the scope of business and expand the scale of the enterprise. This may result in asymmetric internal information and increase the difficulty of management decision-making. Diversification of enterprises requires entry into new industries. Enterprises need a lot of capital and resources when facing high trade barriers, and external financing will inevitably bring about enterprise liabilities (Sharma et al., 2021). When choosing a new industry, managers have an increased rate of decision-making errors due to a lack of information superiority. This may cause the situation that the investment in the new industry is not as good as the income. The business risk of the enterprise increases. So diversification model may reduce enterprise performance (Wu et al., 2017).

### The Relationship Between the Diversified Operation of Media Enterprises and Entrepreneurship

Entrepreneurship has a direct and indirect influence on the diversified operation of media enterprises. (1) Direct impact: if media enterprises pay more attention to entrepreneurship, the enterprise will produce a positive characteristic in the overall atmosphere. In addition, enterprises with entrepreneurship have a greater sense of social responsibility. Generally, such enterprises will analyze and meet their needs from society’s and customers’ perspectives and create greater social value. (2) Indirect impact: entrepreneurship can improve the market position of enterprises through indexes. Entrepreneurship will affect the enterprises’ growth with other variables such as organizational learning and the external environment (Kijkasiwat and Phuensane, 2020).

Equation (3) is the calculation method to measure the financial performance of enterprises.


(3) ROE=S/P

*ROE* represents the ROE, *S* represents the net profit, and *P* represents the total assets.

Equation (4) is the calculation method of sales revenue growth rate.


(4) L=(A−B)/B

In Equation (4), *L* represents the growth rate of sales revenue, *A* represents the sales revenue of the current period, and *B* represents the sales revenue of the previous period.

Equation (5) is the calculation method of market share.


(5) Z=T−H

In Equation (5), *Z* represents the market share, *T* represents the operating revenue of the company, and *H* represents the operating revenue of all listed companies in the industry.

Besides, economic value added (EVA) refers to the income after deducting the total invested capital cost including equity and debt from the after-tax net operating income. Equation (6) is the specific calculation method.


(6) EVA=R−C=X+Y−J∗N

In Equation (6), *R* represents the operating income before interest and tax, *C* represents the average cost of invested capital, *X* represents the after-tax profit, *Y* represents the interest, *J* represents the invested cost, and *N* represents the weighted average cost of capital.

### Questionnaire Design of Entrepreneurship

#### Purpose of the Investigation

This paper studied the influence of the media diversification model and entrepreneurship on enterprise financial performance in the environment of sustainable development. Suggestions to improve enterprise financial performance were proposed through the data analysis of the survey results to help enterprises increase their market competitiveness.

#### Object of Investigation

**Figure 7:**
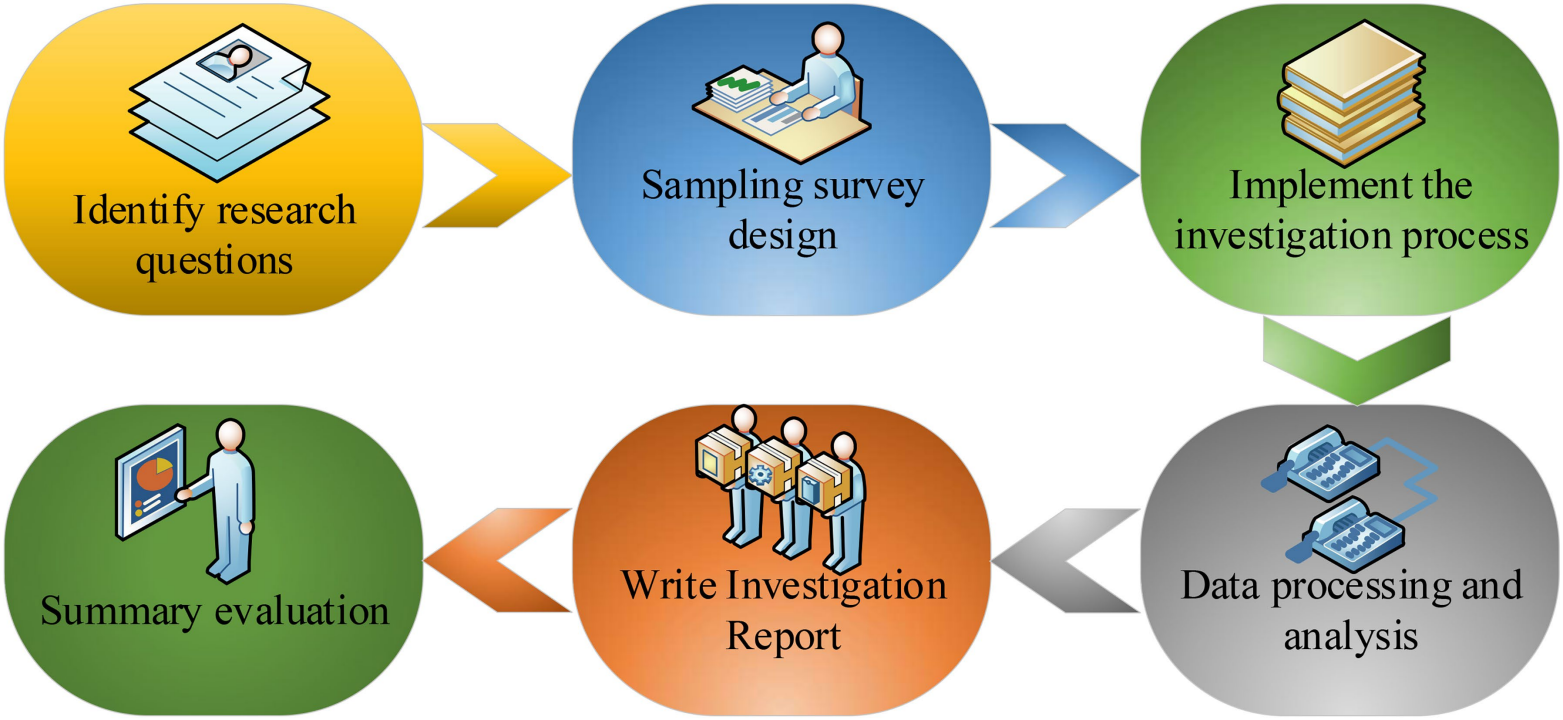
Questionnaire survey steps.

Enterprises in the Yangtze River Delta were the research object. Questionnaires were distributed to 110 randomly selected entrepreneurs. Before the questionnaires were distributed, they were discussed with experts in the relevant professions to ensure the scientific nature of the questionnaires. Finally, the unreasonable places in the questionnaires were revised. The form of face-to-face distribution and on-site recovery was taken to ensure the recovery rate. Ninety-six copies were recovered, the recovery rate was about 87.27%, the effective recovery number was 81, and the effective recovery rate was 84.38%. Figure 7 shows the specific investigation steps.

From Figure 7, the method of questionnaire survey is mainly composed of six parts, which are determining the research question, designing the sampling survey, executing the questionnaire survey, processing and analyzing the data, writing the survey report, and summarizing the results.

Kaiser Meyer Olkin (KMO) validity test is performed on the data to make the results of the questionnaire accurate, as shown in Equation (7).


(7) $$\mathrm{KMO}=\frac{\sum \sum_{i\neq j}r_{ij}^{2}}{\sum \sum_{i\neq j}r_{ij}^{2}+\sum_{i\neq j}r_{ij•1,2\ldots k}^{2}}$$

In Equation (7), *r*, *i*, *j*, and *k* represent the correlation coefficient, dependent variable, independent variable, and quantity, respectively. Table 1 displays the specific test criteria.

**Table 1:** Kaiser Meyer Olkin (KMO) test criteria.

Type	Range of values	Factor analysis is appropriate
KMO value	>0.9	Very much suitable
	0.8–0.9	Quite suitable
	0.7–0.8	Fit
	0.6–0.7	Not very suitable
	0.5–0.6	Barely fit
	<0.5	Unsuited

The SPSS25.0 is used to analyze the validity of the designed questionnaire data. The KMO value is 0.869, which is between 0.8 and 0.9, and (*p* = 0) < 0.01. The results show that the questionnaire data are appropriate for factor analysis, and the questionnaire has good validity.

Moreover, the reliability test is carried out on the data of the questionnaire survey, and the specific calculation method is shown in Equation (8).


(8) $$\alpha =\frac{k}{k-1}\left(1-\frac{\sum_{i=1}^{k}\sigma_{Yi}^{2}}{\sigma_{X}^{2}}\right)$$

In Equation (8), *k* represents quantity. *X* represents the dependent variable. *Y* represents the independent variable. Generally speaking, the higher the reliability coefficient, the higher the reliability between variables, indicating the higher the degree of internal consistency between variables. The internal consistency reliability test of the responses to the questionnaire is carried out through the above equation, and the calculated result is 0.86, indicating that the reliability of the questionnaire is high.

## Analysis of the Effect of the Diversification Model and Entrepreneurship on Financial Performance

### The Impact of the Media Diversification Model on Financial Performance

**Figure 8:**
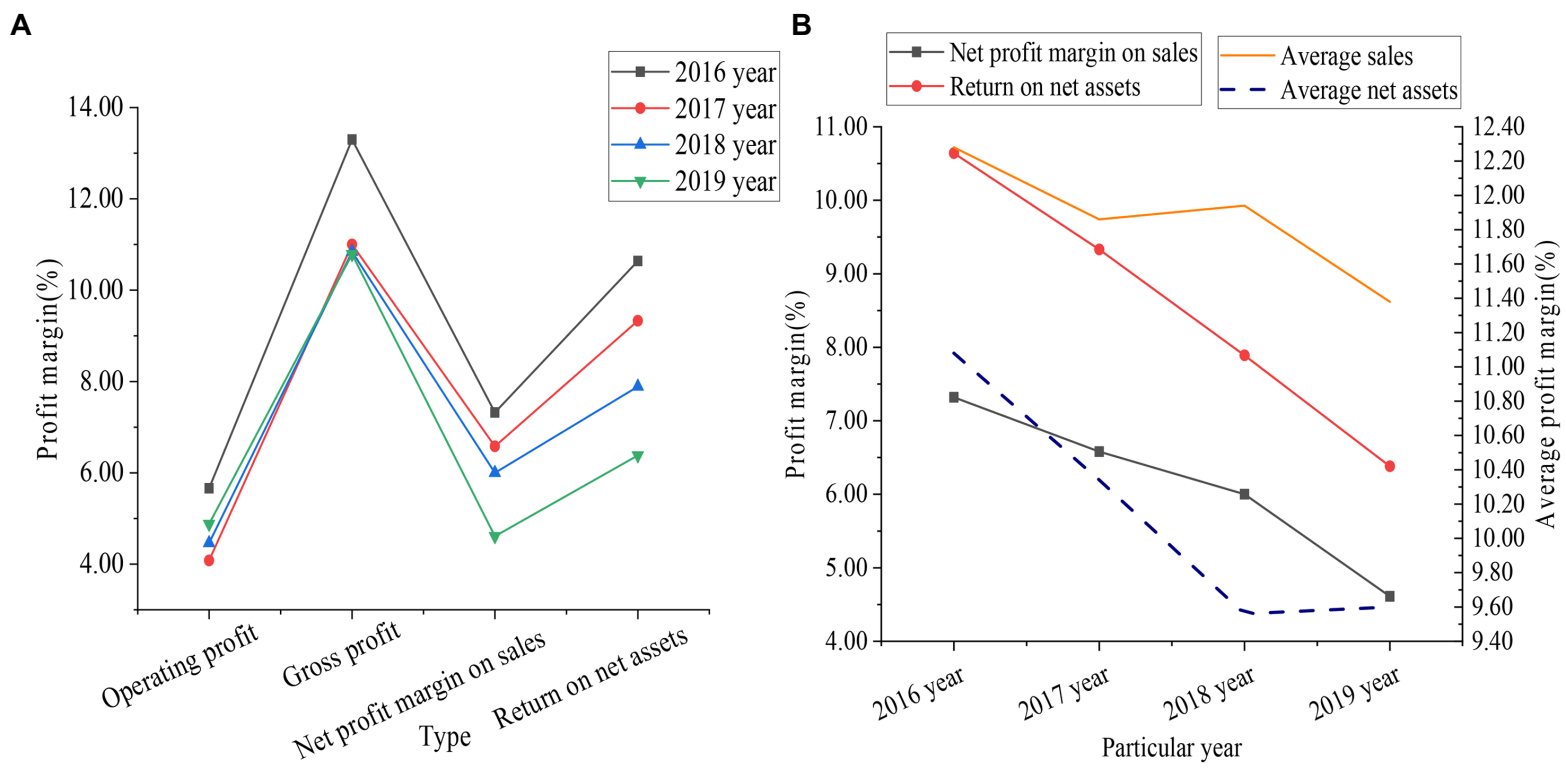
Earnings index data of Time Publishing & Media from 2016 to 2019 (**A** shows the specific profit data of the enterprise, and **B** shows the comparison results between the enterprise and the average industry profit).

The influence of the media diversification model on enterprise financial performance was deeply studied in the environment of sustainable development. Time Publishing & Media was taken as the research object. The method of questionnaire survey was used. Its profit data from 2016 to 2019 are demonstrated in Figure 8.

From Figure 8, the operating profit, gross profit of sales, net profit of sales, and ROE of Time Publishing & Media continued to decline from 2016 to 2019. Operating profit decreased by 0.78%, gross profit from sales decreased by 2.51%, net profit from sales decreased by 2.71%, and ROE decreased by 4.26%. Among the four sets of data, ROE declined the most. From Figure 8B, the net sales profit margin of Time Publishing & Media was 7.32% in 2016 and 4.61% in 2019. The industry average net sales profit margin was 12.28% in 2016 and 11.38% in 2019. The ROE of Time Publishing & Media was 10.64% in 2016 and 6.38% in 2019. The industry average ROE was 11.08% in 2016 and 9.60% in 2019. According to these data, the net sales margin and ROE of Time Publishing & Media are far from the industry average. This reveals that Time Publishing & Media’s profitability is weak, and the diversified management methods do not contribute to profitability improvement.

**Figure 9:**
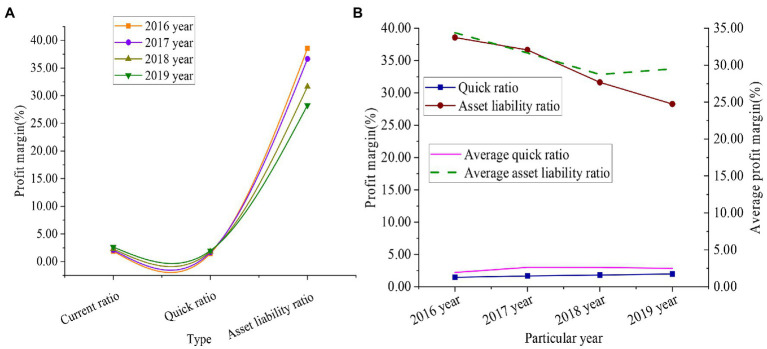
Debt service index data of Time Publishing & Media from 2016 to 2019 (**A** shows the specific debt repayment data of the enterprise, and **B** shows the comparison results between the enterprise and the industry average debt repayment).

Figure 9 shows the debt service index data of Time Publishing & Media from 2016 to 2019.

Figure 9 shows that the current ratio of Time Publishing & Media increased by 0.77%, the quick ratio increased by 0.52%, and the asset-liability ratio decreased by 10.30%. The industry average current ratio was 2.21% in 2016, and 2.87% in 2019, an increase of 0.66%. The industry average quick ratio was 1.94% in 2016 and 2.50% in 2019, increasing 0.56%. The industry’s average asset-liability ratio was 34.40% in 2016 and 29.50% in 2019, a decrease of 4.90%. It reveals that the long-term solvency of Time Publishing & Media is relatively good, and the risk of debt repayment is low.

**Figure 10:**
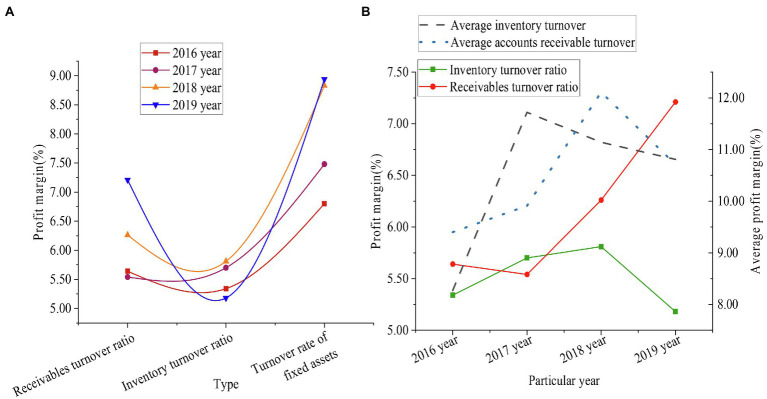
Operation capacity index data of Time Publishing & Media (**A** shows the specific operation data of the enterprise, and **B** shows the comparison results between the enterprise and the average operation of the industry).

In addition, Figure 10 shows the operation capacity evaluation data of Time Publishing & Media from 2016 to 2019.

From Figure 10, the accounts receivable turnover rate of Time Publishing & Media increased by 1.56%, the inventory turnover rate decreased by 0.16%, and the fixed asset turnover rate increased by 2.14%. It reflected that the turnover speed of Time Publishing & Media’s assets accelerated, the enterprise’s ability to sell goods enhanced, and the overall operational efficiency and effectiveness of the enterprise improved. The industry average inventory turnover ratio in 2016 was 8.27%, and in 2019 it was 10.81%, by 2.54%. The industry accounts receivable turnover ratio was 9.40% in 2016 and 10.71% in 2019, by 1.31%. Compared with the average level of the industry, although Time Publishing & Media improved their operating capabilities, the industry averages were different. This requires Time Publishing & Media to strengthen the management of accounts receivable and inventory.

### The Influence of Entrepreneurship on Financial Performance

**Figure 11:**
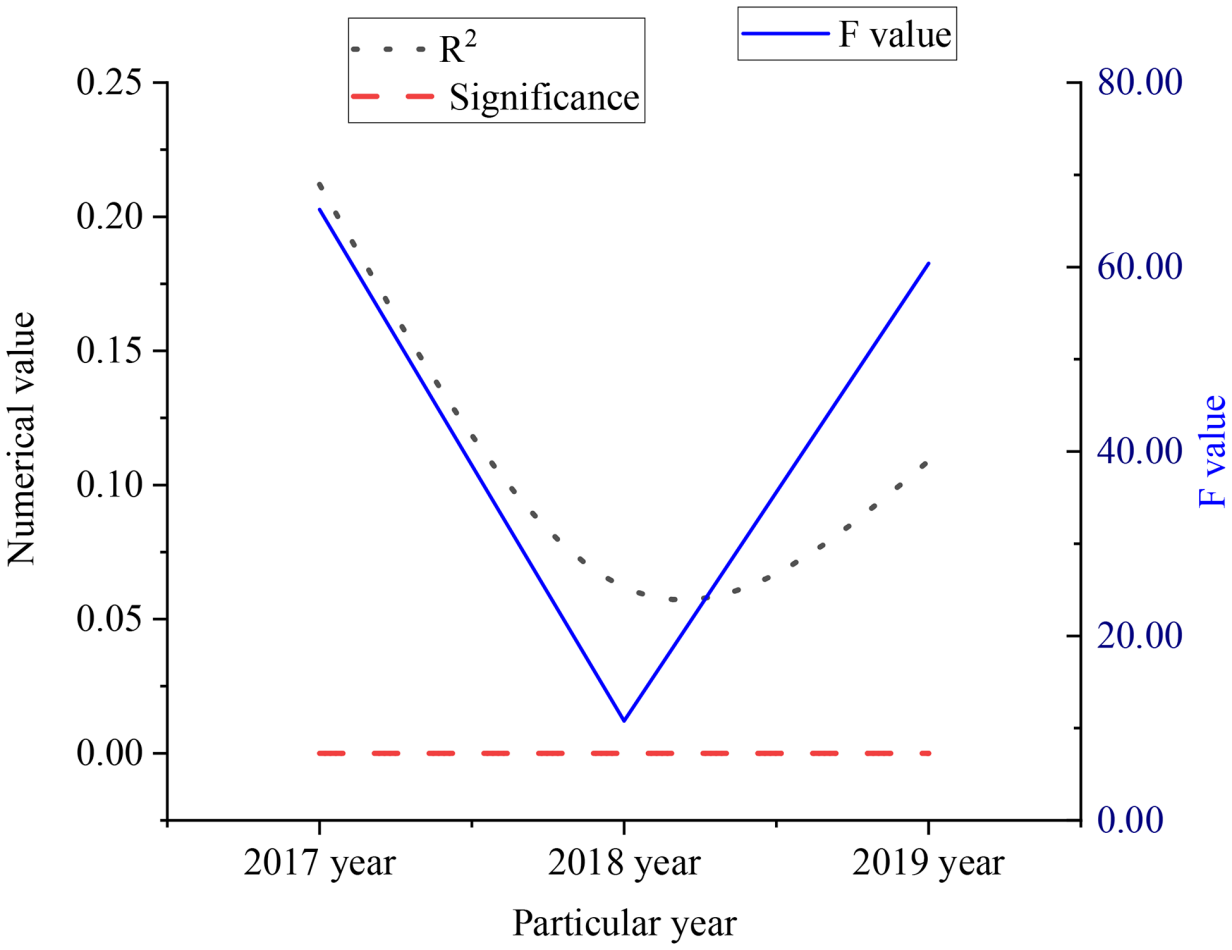
Linear regression data of questionnaire.

The above questionnaire method is used to obtain relevant data about entrepreneurs in the Yangtze River Delta, and conduct linear regression analysis with enterprise financial performance, as shown in Figure 11.

From Figure 11, the values of R2 in 2017–2019 are about 0.21, 0.06, and 0.11, respectively. The *F* values in 2017–2019 are about 66.24, 10.78, and 60.39, respectively. The significance of values is 0.00. As for enterprise financial performance, the capital expenditures undertaken by entrepreneurs from 2017 to 2019 had the greatest impact on enterprise financial performance. According to this situation, entrepreneurial risk-taking spirit and financial performance are related. The stronger the entrepreneur’s risk-taking spirit, the better the financial performance of the enterprise. However, the degree of this risk is not as high as possible. If the risk is too high, bankruptcy will occur.

## Conclusion

With the deepening of economic globalization, market competition has become increasingly intense. The influence of the media diversification model and entrepreneurship on enterprise financial performance was analyzed under the environment of sustainable development. Time Publishing & Media were taken as the representatives of the media diversification model. The questionnaire survey and comparative analysis were the research methods. The following are conclusions: (1) Time Publishing & Media is taken as the research object. Its profit data from 2016 to 2019 are studied and compared with the industry average. It is found that the net profit margin on sales and return on net assets of Time Publishing & Media are far from the industry average, which shows that the profitability of Time Publishing & Media is weak to a certain extent, and the diversified operation mode has not achieved the improvement of profitability. (2) Compared with the industry level, Time Publishing & Media has better long-term solvency and lower debt repayment risk. (3) Although the operation capacity of Time Publishing & Media has increased to a certain extent, it is different from the average value of the same industry. It requires Time Publishing & Media to strengthen the management of accounts receivable and inventory. (4) Meanwhile, the questionnaire survey method is used to analyze the relationship between entrepreneurship and corporate finance. The results show that there is a positive correlation between entrepreneurs’ risk-taking ability and enterprise financial performance. However, the ability to bear risks should also be consistent with the actual social situation. If the risk level is too high, it may not obtain better financial performance.

There are limitations in data acquisition due to limited time. It leads to errors in some tests of the relevant data. In addition, the influence of the media diversification model and entrepreneurship on enterprise financial performance in the environment of sustainable development has not been discussed in terms of economic cost input. Benefit evaluation will be carried out on a case-by-case basis in the future to obtain excellent financial performance for enterprises.

## Data Availability Statement

The raw data supporting the conclusions of this article will be made available by the authors, without undue reservation.

## Ethics Statement

The studies involving human participants were reviewed and approved by Jeonju University Ethics Committee. The patients/participants provided their written informed consent to participate in this study. Written informed consent was obtained from the individual(s) for the publication of any potentially identifiable images or data included in this article.

## Author Contributions

All authors listed have made a substantial, direct, and intellectual contribution to the work and approved it for publication.

## Conflict of Interest

The authors declare that the research was conducted in the absence of any commercial or financial relationships that could be construed as a potential conflict of interest.

## Publisher’s Note

All claims expressed in this article are solely those of the authors and do not necessarily represent those of their affiliated organizations, or those of the publisher, the editors and the reviewers. Any product that may be evaluated in this article, or claim that may be made by its manufacturer, is not guaranteed or endorsed by the publisher.
